# Atrial Fibrillation Ablation Using Robotic Magnetic Navigation Reduces the Incidence of Silent Cerebral Embolism

**DOI:** 10.3389/fcvm.2021.777355

**Published:** 2021-12-01

**Authors:** Jie Zheng, Meng Wang, Qun-feng Tang, Feng Xue, Ku-lin Li, Shi-peng Dang, Xiao-yu Liu, Xiao-xi Zhao, Chang-ying Zhang, Zhi-ming Yu, Bing Han, Ting-bo Jiang, Yan Yao, Ru-Xing Wang

**Affiliations:** ^1^Department of Cardiology, Wuxi People's Hospital Affiliated to Nanjing Medical University, Wuxi, China; ^2^Department of Cardiology, Xuzhou Central Hospital, Xuzhou, China; ^3^Department of Radiology, Wuxi People's Hospital Affiliated to Nanjing Medical University, Wuxi, China; ^4^Department of Cardiology, The First Hospital Affiliated to Soochow University, Suzhou, China; ^5^Department of Cardiology, Fuwai Hospital, Chinese Academy of Medical Sciences - Peking Union Medical College, Beijing, China

**Keywords:** silent cerebral embolism, atrial fibrillation, ablation technology, robotic magnetic navigation, catheter ablation

## Abstract

**Background:** The incidence of silent cerebral embolisms (SCEs) has been documented after pulmonary vein isolation using different ablation technologies; however, it is unreported in patients undergoing with atrial fibrillation (AF) ablation using Robotic Magnetic Navigation (RMN). The purpose of this prospective study was to investigate the incidence, risk predictors and probable mechanisms of SCEs in patients with AF ablation and the potential impact of RMN on SCE rates.

**Methods and Results:** We performed a prospective study of 166 patients with paroxysmal or persistent AF who underwent pulmonary vein isolation. Patients were divided into RMN group (*n* = 104) and manual control (MC) group (*n* = 62), and analyzed for their demographic, medical, echocardiographic, and risk predictors of SCEs. All patients underwent cerebral magnetic resonance imaging within 48 h before and after the ablation procedure to assess cerebral embolism. The incidence and potential risk factors of SCEs were compared between the two groups. There were 26 total cases of SCEs in this study, including 6 cases in the RMN group and 20 cases in the MC group. The incidences of SCEs in the RMN group and the MC group were 5.77 and 32.26%, respectively (X^2^ = 20.63 *P* < 0.05). Univariate logistic regression analysis demonstrated that ablation technology, CHA_2_DS_2_-VASc score, history of cerebrovascular accident/transient ischemic attack, and low ejection fraction were significantly associated with SCEs, and multivariate logistic regression analysis showed that MC ablation was the only independent risk factor of SCEs after an AF ablation procedure.

**Conclusions:** Ablation technology, CHA_2_DS_2_-VASc score, history of cerebrovascular accident/transient ischemic attack, and low ejection fraction are associated with SCEs. However, ablation technology is the only independent risk factor of SCEs and RMN can significantly reduce the incidence of SCEs resulting from AF ablation.

**Clinical Trial Registration:** ChiCTR2100046505.

## What's New?

- This is the first study comparing AF ablation using robotic magnetic navigation with manual control, showing significant reduction in the incidence of silent cerebral embolisms after ablation procedure.
- Ablation technology, CHA_2_DS_2_-VASc score, CVA/TIA history, and low ejection fraction are associated with silent cerebral embolisms in AF ablation patients, and among these factors, ablation technology is the only independent risk factor of silent cerebral embolisms.
- The mechanisms of decreased incidence of silent cerebral embolisms in AF ablation using robotic magnetic navigation may be due to the reduction of gaseous microbubbles, clot formation and char formation.

## Introduction

Atrial fibrillation (AF) is the most common sustained cardiac arrhythmia with an increasing prevalence that affects at least 1% of the population worldwide and is associated with increased morbidity and mortality (1). AF catheter ablation with the goal of pulmonary vein isolation (PVI) has been established as an important therapeutic option for the treatment of AF. However, the complexity of the procedure may expose patients to a considerable number of complications. Stroke and thromboembolism are among the most harmful periprocedural complications following AF ablation procedures (2). Although symptomatic cerebral embolisms are rare (<1%) during PVI procedures (3, 4), new silent cerebral embolisms (SCEs) detected by cerebral magnetic resonance imaging (MRI) scans have a reported incidence of >10%, with some publications reporting SCEs in up to 30% of patients (5, 6). The long-term clinical significance of SCEs remains unclear, however they may correlate with neurologic deficits, including an increased risk of dementia. Therefore, it is imperative to take measures to reduce the risk of SCEs.

The issue of SCEs developing perioperatively in AF patients undergoing catheter ablation was first brought to general attention by Lickfett et al. (7) in 2006. Several mechanisms have been suggested as being potentially responsible for SCEs after AF catheter ablation, including macrobubble development within stationary sheaths, radiofrequency- and heat- related denaturation of fibrinogen to fibrin, and catheter manipulation in the left atrium (8). In the last decade, the exploration of SCE mechanisms has been focused on the risk of different AF ablation technologies and new ablation tools, which have various effects on the incidence of SCEs (9, 10). A high incidence of SCEs has been reported recently using the duty-cycled phased-radiofrequency ablation tool (6).

Robotic navigation technology has emerged as an important new tool to facilitate catheter ablation of arrhythmias (11). Mechanical robotic navigation has previously been shown to have a similar rate of SCEs compared to manual control (MC) ablation which we hypothesize is due to the similar manual pull-wire catheter technology employed (12). Robotic magnetic navigation (RMN) employs a fundamentally different mechanism of action. Direct manipulation of the catheter tip using magnetic fields allows for the elimination of pull-wires and braided shafts making the catheter body extremely soft and flexible providing a gentle interface with tissue. The enhanced steerability enables reaching targets without extensive sheath manipulation (13–15).

Numerous studies have shown RMN to be as effective as MC ablation (16–18), while reducing periprocedural complications and fluoroscopy exposure (19), but incidence of SCEs has not been reported. The purpose of this prospective pilot study was to examine the incidence of SCEs and investigate the potential risk predictors and possible mechanisms of SCEs in AF patients undergoing RMN-assisted PVI.

## Methods

### Patient Population

In this multi-center prospective pilot study (Registered Clinical Trial Number: ChiCTR2100046505), a total of 166 AF patients, including 110 paroxysmal and 56 persistent AF patients, 100 males and 66 females, with mean age of 61.03 ± 9.58 (25-79) years, were enrolled for AF ablation at Wuxi People's Hospital affiliated to Nanjing Medical University (Wuxi, China), Xuzhou Central Hospital (Xuzhou, China), and the First Hospital affiliated to Soochow University (Suzhou, China). In these three centers, 104 AF patients were ablated using RMN at Wuxi People's Hospital affiliated to Nanjing Medical University (RMN group), and 62 AF patients were ablated using MC ablation at Xuzhou Central Hospital and the First Hospital affiliated to Soochow University (MC group). All patients were undergoing their first AF ablation procedure and no redo cases were included in this study.

The criteria of inclusion and exclusion, and the definitions of paroxysmal AF and persistent AF were the same as we previously reported (14). In brief, paroxysmal AF was defined as self-terminating within 7 days or terminated with electrical or pharmacological cardioversion. Persistent AF was defined as lasting >7 days, requiring cardioversion or other intervention or failed cardioversion, or cardioversion was no longer attempted. All patients provided written informed consent prior to study enrollment. The study protocol was reviewed and approved by the ethics committees of all three centers.

### Electrophysiological Study and Catheter Ablation

AF ablation using RMN in 104 patients was performed as we previously reported (14). Procedures in the RMN group were performed using the Niobe Magnetic Navigation System (Stereotaxis, Inc., St Louis, MO, USA) paired with a Siemens Axiom Artis fluoroscopy system (Siemens, Erlangen, Germany). Atrial septum puncture was performed only once and one Swartz SL1 sheath was placed into the left atrium. The third-generation irrigated magnetic catheters (Navistar RMT Thermocool; Biosense Webster, Inc. Diamond Bar, California) were steered omni-directionally by a magnet field-controlled system which follows the direction of applied vectors, and were advanced or retracted in 1 to 9 mm steps by a mechanical device (QuikCAS™ automated catheter advancement system, Stereotaxis, St. Louis, MO, USA). Ablation was performed remotely from the control room, away from radiation exposure, utilizing the computer mouse and keyboard of the RMN.

The AF ablation procedures in 62 AF patients with MC technology were performed using the commonly used technologies and methods described by Haissaguerre and colleagues previously. In brief, after placing a diagnostic catheter into the coronary sinus, the atrial septum was punctured twice and two Swartz SL1 sheaths were placed into the left atrium. An irrigated-tip contact force ablation catheter (Thermocool SmartTouch, Biosense Webster, Diamond Bar, CA, USA) was used to perform the ablation. Radiofrequency energy was applied in a power-controlled mode with a power limit of 30–35 W and a maximum temperature of 43°C, which is the same as AF ablation procedure using RMN. In the process of the entire AF ablation procedure, the two sheaths were constantly being moved backward and forward, especially the sheath for ablation. Catheter exchanges from one sheath to the other sheath were often performed to facilitate the catheter ablation of pulmonary vein isolation.

Only circumferential PVI was performed in patients with paroxysmal AF. In patients with persistent AF, linear ablation of the left atrial roof and mitral isthmus was performed in addition to circumferential PVI. Electrical cardioversion was carried out for persistent AF patients with failure to maintain sinus rhythm after AF ablation procedures. During the entire AF ablation procedure, the sheath was fixed to the mechanical device (QuikCAS™) and almost no catheter exchanges were needed.

Left atrial thrombi in all enrolled patents were ruled out by transesophageal echocardiography 24 h before the ablation procedure. All procedures were performed under deep sedation using boluses of midazolam, fentanyl and a continuous infusion of propofol.

### Periprocedural Anticoagulation

Warfarin or new oral anticoagulants (Dabigatran or Rivaroxaban) were taken orally no <3 weeks prior to the ablation procedure and taken for 2 months after ablation in all patients. If the patients chose warfarin as an oral anticoagulation, an international normalized ratio (INR) ≥2.0 was achieved and initiated ≥3 weeks before the procedures. If the patients chose Dabigatran or Rivaroxaban, we did not perform the routine monitoring of INR. After atrial septal puncture, heparin was immediately injected as a bolus of 70–100 I.U. per kg body weight from a peripheral vein. Heparin was administered later through the cooling fluid of the ablation catheter as well as via intermittent venous injections. The activated clotting time (ACT) was measured every 20–30 min to maintain 250 to 300 s during the ablation procedures. Oral anticoagulation was maintained throughout the ablation procedures without interruption or bridging and for the duration of follow-up.

### Diffusion-Weighted MRI

A cerebral MRI was performed within 48 h before and after each PVI procedure using GE Sigma Excite 1.5 or 3.0 T scanners (Magnetom Aera, Siemens Healthcare, Erlangen, Germany or Magnetom Trio, Siemens Medical Solution, Erlangen, Germany) as previously reported (20). Diffusion-weighted MRI (DW-MRI) images from before and after the AF ablation procedure were analyzed and compared to identify any procedure-related acute cerebral lesions. Acute cerebral lesions were defined as the presence of focal diffusion abnormalities (bright hyper-intense lesions) in either a cortical or subcortical location, or in the vascular territory of the perforating arteries. The location and sizes of focal diffusion abnormalities were analyzed. Locations were categorized as: (1) left or right hemisphere and (2) locations within the (a) the frontal lobe, (b) parietal lobe, (c) temporal lobe, (d) precentral gyrus, (e) occipital lobe, (f) postcentral gyrus, or (g) cerebellum. The sizes of SCEs were defined as small (≤ 3 mm maximum diameter), medium (3 to 10 mm) and large (≥10 mm). In the case of differing DW-MRI readings, a consensus was obtained. All DW-MRI data, including number, location, and size of embolic lesions, were analyzed independently by two experienced radiologists who were blinded to the study data. No differences in evaluation, classification, or quantitative results were noted between the two experts.

### Statistical Analysis

All continuous data were checked for normality with the Kolmogorov-Smirnov test and the Levene test was used for homogeneity of variance. Normally distributed continuous variables are described as mean ± standard deviations ($\bar{X}$ ± SD). Categorical variables are described as median with inter-quartile range. The data distribution was compared using either the Student *t*-test or Mann-Whitney U test. For categorical data, the count and percentages were provided and compared by using a Chi-square test or Fisher exact test for low expected count. A logistic regression analysis was performed to investigate the relationship between SCEs and the baseline or procedural characteristics. The variables with either *P* < 0.05 or important clinical variables, such as diabetes and high score in the univariate analysis were selected for testing in the multivariate analysis. All tests were 2-sided, and statistical significance was set at a value of *P* < 0.05. Statistical analysis was carried out using SPSS 22.0 (IBM Corporation, Armonk, New York, USA).

## Results

### Patient Characteristics

One hundred and sixty six patients including 104 in the RMN group and 62 in MC group were enrolled and ablated in the study. Differences were noted with respect to several baseline characteristics, including age, CHA_2_DS_2_-VASc score, cardioversion, left atrial diameter, ejection fraction, heart function class, and history of cerebrovascular accident/transient ischemic attack (CVA/TIA). No remarkable distinction was found in sex, body mass index, AF type, AF duration, ACT during procedure, or comorbidities such as hypertension, coronary artery disease, diabetes mellitus, and dislipidemia. The baseline clinical and demographic characteristics of the study population are detailed in Table 1.

**Table 1:** Baseline clinical and demographic characteristics of the study population.

**Variables**	**All population (*n* = 166)**	**RMN group (*n* = 104)**	**MC group (*n* = 62)**	***P-*value**
Sex (M/F)	100/66	65/39	35/27	0.441
Age (y)	61.03 ± 9.58	59.06 ± 10.10	64.34 ± 7.65	0.000
BMI (kg/m^2^)	24.64 ± 2.57	24.50 ± 2.34	24.88 ± 2.96	0.404
**AF type**				
Paroxysmal AF/Persistent AF	110/56	70/34	40/22	0.713
AF duration (m)	24	24	30	0.445
CHA_2_DS_2_-VASc score	2.48 ± 1.58	2.12 ± 1.36	3.08 ± 1.74	0.000
Cardioversion (Y/N)	56/110	29/75	27/35	0.039
ACT during procedure (s)	285.90 ± 14.94	285.12 ± 14.57	287.23 ± 15.58	0.380
LA diameter (mm)	37.93 ± 5.76	38.97 ± 5.16	36.19 ± 6.31	0.002
EF (%)	60.67 ± 5.68	62.50 ± 4.73	57.60 ± 5.84	0.000
Heart Function Class (NYHA)	1.16 ± 0.38	1.23 ± 0.45	1.03 ± 0.18	0.001
**Comorbidity (** ***n*** **)**				
HTN	76	43	33	0.137
CAD	34	26	8	0.062
DM	19	10	9	0.337
Dyslipidemia	4	4	0	0.298
Hyperthyroidism	5	1	4	0.065
CVA/TIA history	8	1	7	0.005
Others	13	5	8	0.076
Absence of underlying diseases (n)	44	26	18	0.569
**Lab examinations**				
BUN (mmol/L)	5.35 ± 1.52	5.09 ± 1.53	5.79 ± 1.41	0.004
Cr (mmol/L)	72.53 ± 18.76	80.72 ± 17.19	58.80 ± 12.14	0.000
FBG (mmol/L)	5.29 ± 1.44	5.15 ± 1.49	5.51 ± 1.34	0.117
TC (mmol/L)	4.24 ± 0.93	4.25 ± 0.88	4.23 ± 1.00	0.897
LDL (mmol/L)	2.41 ± 0.81	2.27 ± 0.78	2.64 ± 0.83	0.004
TG (mmol/L)	1.89 ± 1.36	2.08 ± 1.57	1.56 ± 0.81	0.046

*The data are presented as the mean ± SD or n (%). RMN, robotic magnetic navigation; MC, manual control; BMI, body mass index; AF, atrial fibrillation; ACT, activated clotting time; LA diameter, left atrial diameter; Y, yes; N, no; HTN, hypertension; CAD, coronary artery disease; DM, diabetes mellitus; CVA, cerebrovascular accident; TIA, transient ischemic attack; BUN, blood urea nitrogen; Cr, creatinine; FBG, fasting blood glucose; TC, total cholesterol; LDL, low density lipoprotein; TG, triglyceride; Absence of underlying diseases, refers to patients with no other cardiovascular co-morbidity or no other co-morbidities at all*.

### Patients With or Without Silent Cerebral Embolism

Additionally, we compared the characteristics of patients with SCE and without SCE. We found that there were significant differences in CHA_2_DS_2_-VASc score, ejection fraction, CVA/TIA history, and creatinine between two groups. There were no differences in sex, age, body mass index, AF type and other variables. The detailed characteristics of patients with SCE and without SCE are demonstrated in Table 2.

**Table 2:** Characteristics of patients with SCE and without SCE.

**Variables**	**All population (*n* = 166)**	**SCE group (*n* = 26)**	**No SCE group (*n* = 140)**	***P-*value**
Sex (M/F)	100/66	12/14	88/52	0.110
Age (years)	61.03 ± 9.58	61.96 ± 8.92	60.86 ± 9.72	0.591
BMI (kg/m^2^)	24.64 ± 2.57	25.52 ± 2.79	24.44 ± 2.49	0.057
**AF type**				
Paroxysmal AF/Persistent AF	110/56	017/9	93/47	0.918
AF duration time (month)	24	24	24	0.365
CHA_2_DS_2_-VASc score	2.48 ± 1.58	3.31 ± 1.64	2.32 ± 1.52	0.003
Cardioversion (Y/N)	56/110	10/16	46/94	0.579
ACT during procedure (seconds)	285.90 ± 14.94	289.96 ± 15.73	285.15 ± 14.73	0.132
LAD (mm)	37.93 ± 5.76	36.08 ± 6.49	38.28 ± 5.57	0.073
EF (%)	60.67 ± 5.68	58.62 ± 5.75	61.05 ± 5.60	0.044
Heart Function Class (NYHA)	1.16 ± 0.38	1.08 ± 0.27	1.17 ± 0.40	0.251
**Comorbidity (** ***n*** **)**				
HTN	76	14	62	0.369
CAD	34	4	30	0.603
DM	19	3	16	1.000
Dyslipidemia	4	0	4	1.000
Hyperthyroidism	5	2	3	0.128
CVA/TIA history	8	4	4	0.022
Others	13	2	11	0.977
Absence of underlying diseases (n)	44	7	37	1.000
**Lab examinations**				
BUN (mmol/L)	5.35 ± 1.52	5.47 ± 1.48	5.33 ± 1.53	0.669
Cr (mmol/L)	72.53 ± 18.76	64.59 ± 17.75	74.01 ± 18.63	0.018
FBG (mmol/L)	5.29 ± 1.44	5.58 ± 1.01	5.23 ± 1.50	0.254
TC (mmol/L)	4.24 ± 0.93	4.14 ± 0.95	4.26 ± 0.92	0.541
LDL (mmol/L)	2.41 ± 0.81	2.43 ± 0.75	2.40 ± 0.83	0.866
TG (mmol/L)	1.89 ± 1.36	1.85 ± 0.95	1.89 ± 1.42	0.893

*The data are presented as the mean ± SD or n (%). LAD, left atrial diameter; EF, ejection fraction; HTN, hypertension; CAD, coronary artery disease; DM, diabetes mellitus; BUN, blood urea nitrogen; Cr, creatinine; FBG, fasting blood glucose; TC, total cholesterol; LDL, low density lipoprotein; TG, triglyceride; CVA, cerebrovascular accident; TIA, transient ischemic attack; Y, yes; N, no; Absence of underlying diseases, refers to patients with no other cardiovascular co-morbidity or no other co-morbidities at all*.

### Incidence, Localization, Number, and Size of Silent Cerebral Embolism

**Figure 1:**
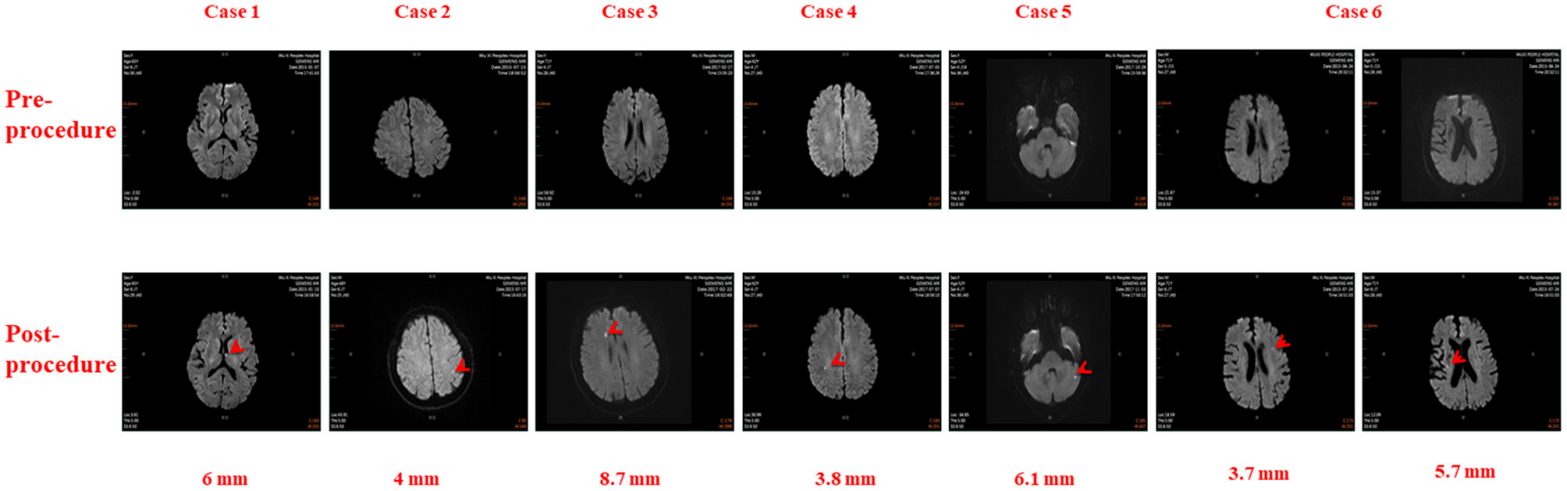
Cerebral Magnetic Resonance Images in six patients before and after Atrial Fibrillation Ablation using Magnetic Navigation System: Case 1: Left thalamus (6 mm), Case 2: Left parietal lobe (4 mm), Case 3: Right frontal lobe (8.7 mm), Case 4: Right parietal lobe (3.8 mm), Case 5: Left cerebellum (6.1 mm), Case 6: Left frontal lobe (3.7 mm) and Right corona radiata (5.7 mm).

There were 26 total cases of SCE observed in this study, including 6 in the RMN group and 20 in the MC group. The incidences of SCE in the RMN group and MC group were 5.77 and 32.26%, respectively (X^2^ = 20.63 *P* < 0.001). The cerebral MRIs of SCEs in the 6 cases observed in the RMN group are shown in Figure 1, and the cerebral MRIs for the 20 cases of SCE in the MC group are provided in the Supplementary Data file.

Localization, number and size of SCEs in all 26 patients are annotated in Table 3. There were 32 total SCEs including 1 (3.13%) patient with multiple cerebral embolisms in the RMN group and 4 (12.50%) patients with multiple cerebral embolisms in the MC group (X^2^ = 0.012, *P* > 0.99). Among the 32 cerebral embolisms, 9 (28.13%) were located in frontal lobes (4 in right frontal lobes and 5 in left frontal lobes), 8 (25.00%) in occipital lobes (4 each in left and right occipital lobes respectively), 5 (15.63%) in the parietal lobes (3 in left parietal lobes and 2 in right parietal lobes), 2 (6.25%) in right corona radiata and one (3.13%) each in the left thalamus, right insular lobe, and left basal ganglia. No SCEs were found in the temporal lobe, precentral/postcentral gyrus, or left/right hemisphere.

**Table 3:** Localization, number and size of SCE in 26 patients.

**Case**	**Group**	**Location**	**Size (mm)**
1	RMN	Left thalamus	6
2	RMN	Left parietal lobe	4
3	RMN	Right frontal lobe	8.7
4	RMN	Right parietal lobe	3.8
5	RMN	Left cerebellum	6.1
6	RMN	Left frontal lobe/Right corona radiata	3.7/5.7
7	MC	Left occipital lobe	13
8	MC	Left frontal lobe	3.5
9	MC	Right parietal lobe	8.6
10	MC	Left cerebellum	7.3
11	MC	Right occipital lobe	3.6
12	MC	Right frontal lobe	1.9
13	MC	Right frontal lobe	3.6
14	MC	Left occipital lobe	9.3
15	MC	Left frontal lobe	6.5
16	MC	Right cerebellum	4.2
17	MC	Left frontal lobe/Right occipital lobe/Left occipital lobe	2.9/2.9/2.6
18	MC	Right insular lobe/Left frontal lobe	2.8/9.6
19	MC	Right frontal lobe	4.5
20	MC	Left occipital lobe	3
21	MC	Bilateral cerebellum	4.2/2.4
22	MC	Right occipital lobe	9.3
23	MC	Right parietal lobe/Left basal ganglia	5.2/10.8
24	MC	Right corona radiata	5
25	MC	Right occipital lobe	3
26	MC	Left parietal lobe	4

*RMN, robotic magnetic navigation; MC, manual control*.

The sizes of cerebral embolisms were 5.37±2.81 (1.90–13.00) mm. Of the 32 SCEs, 8 (25.00%) were of small size (≤ 3 mm), 22 SCEs (68.75%) were medium size (3–10 mm), and 2 (6.25%) were large size (≥10 mm). There was no difference in sizes between the RMN and MC groups [5.43 ± 1.79 (3.70–8.70) mm and 5.35 ± 3.07 (1.90–13.00) mm, respectively (t = 0.090, *P* > 0.05)].

### Risk Predictors of Silent Cerebral Embolism

Univariate logistic analysis demonstrated that ablation technology, CHA_2_DS_2_-VASc score, CVA/TIA history, and low ejection fraction were associated with SCEs (*P* < 0.05), however, multivariate logistic regression analysis demonstrated that ablation technology was the only independent risk factor for SCEs (odds ratio [OR] 7.78, 95% confidence interval [CI] 2.92-20.75, *P* < 0.001) (Table 4). Compared with MC ablation, AF ablation using RMN significantly reduced the incidence of SCE (X^2^ = 20.63 *P* < 0.001).

**Table 4:** Risk factors of silent cerebral embolism after pulmonary vein isolation in atrial fibrillation ablation.

**Variable**	**Univariate analysis**	**Multivariate analysis**			
	***P-value***	**Wald**	**Odds ratio**	**95% CI**	***P-value***
Sex	0.110				
Age	0.588				
BMI	0.120				
AF type	0.918				
AF duration	0.419				
CHA_2_DS_2_-VASc score	0.003				
Cardioversion	0.579				
ACT	0.130				
LA diameter	0.072				
Heart functional class	0.244				
EF	0.044				
Ablation technology	0.000	16.785	7.778	2.915–20.751	0.000
**Underlying diseases**					
HTN	0.369				
CAD	0.483				
DM	0.987				
Dislipidemia	0.383				
Hyperthyroidism	0.128				
CVA/TIA history	0.006				

*BMI, body mass index; AF, atrial fibrillation; ACT, activated clotting time; LA diameter, left atrial diameter; EF, ejection fraction; HTN, hypertension; CAD, coronary artery disease; DM, diabetes mellitus; CVA, cerebrovascular accident; TIA, transient ischemic attack*.

## Discussion

### Main Findings

AF ablation carries a low risk of symptomatic cerebral ischemia but is associated with a substantial risk of SCEs detectable with MRI. The incidence of SCEs after AF ablation is affected by different ablation technologies; however, it has not been previously reported for patients undergoing AF ablation using RMN. To the best of our knowledge this is the first prospective study of this kind. The main findings are that: (1) AF ablation using RMN significantly reduced the incidence of SCEs after AF ablation procedures; (2) Ablation technology, CHA_2_DS_2_-VASc score, CVA/TIA history, and low ejection fraction were associated with SCEs in AF ablation patients; (3) SCEs were mainly located in the frontal lobe, occipital lobe, and parietal lobe; and the sizes of SCEs were not observed to differ significantly between RMN ablation and MC ablation groups.

### Definition and Incidence of Silent Cerebral Embolism

Since the first description of SCEs in patients after AF ablation in 2006 (7), SCEs have been widely reported (5, 21, 22). SCE is now defined as ≥1 cerebral infarctions of hypointense lesions on T1-weighted images and hyperintense lesions on T2-weighted images of cerebral MRI, without a history of corresponding stroke or TIA (21). Incidence of SCEs after AF ablation ranges from 1 to ~40% depending on the different risk factors (23, 24). Gaita et al. (25) assessed the thromboembolic risk with preprocedural and postprocedural cerebral MRI, either silent or clinically manifest, in the context of AF ablation, and found AF ablation carried a low risk of symptomatic cerebral ischemia (0.4%) but was associated with a substantial risk of SCEs (14%) as detected on cerebral MRI. Ichiki et al. (26) investigated the incidence of cerebral thromboembolism after complex fractionated atrial electrogram ablation with or without PVI and found the incidence of cerebral thromboembolism after PVI ranges from 2 to 14%. The prevalence has been reported from 6.8 to 38.4% for patients ablated with irrigated-tip radiofrequency catheters (21, 27). Cerebral thromboembolism can be found using high-resolution DW-MRI after AF ablation (28, 29). In this study, we explored the incidence of SCEs in AF ablation using RMN and found that the incidence of SCEs in the RMN group was only 5.77% as compared to 32.26% in the MC group, suggesting that AF ablation using RMN can significantly reduce the incidence of SCEs after procedures.

### Localization, Number and Size of Silent Cerebral Embolism

FLAIR-MRI usually reveals pre-existing cerebral lesions, while DW-MRI demonstrates new SCEs (28, 29). Therefore, we used DW-MRI to evaluate SCEs in this study, and found SCEs were generally distributed throughout the brain, and often involved in the cerebral cortex and cerebellum (20, 22). In our study, we also found most SCEs were located in the lobes of the cortex, but that SCEs were also observed in the left thalamus, right insular lobe and left basal ganglia. Previous studies have demonstrated that SCEs were generally small in size, and most SCEs observed acutely after AF ablation procedures were ≤ 10 mm in diameter (20, 30). In Deneke et al. (30) 52% of the lesions were small (≤ 3 mm), 42% were medium (4 to 10 mm) and 6% were of large diameter >10 mm. However, in our study, 68.75% of lesions were medium (3 to 10 mm), 25.00% were small (≤ 3 mm), and 6.25% were large (≥10 mm). Furthermore, the sizes of SCEs were almost the same between the two groups. We compared our patients' data and ablation procedure with Deneke et al. and found that CHA2DS2-VASc scores in our population were much higher than those in the Deneke et al. population. Additionally, ACTs in our study were shorter than those in the Deneke et al. study, which may be two important causes for the difference in SCE sizes.

### Potential Risk Factors for Silent Cerebral Embolism

SCE is a potential serious complication during AF ablation and 10 times more common than clinical stroke (31). Though patients with SCEs often experience no immediate neurological symptoms, many studies have demonstrated that incurring cerebral acute lesions as a result of PVI may be associated with longer-term adverse neuropsychological outcomes such as an increased risk of stroke and cognitive impairment (32). Therefore, it is of great clinical importance to investigate the risk factors of SCEs associated with AF ablation in an attempt to reduce SCEs (23).

Schrickel et al. (33) showed that coronary artery disease, left ventricular dilation, and hypertrophy were potential risk predictors of SCEs. Martinek et al. (34) found that a variety of different clinical and procedural factors seemed to contribute to the risk of SCEs. Clinical parameters showing a significant correlation with SCEs in univariate analysis were age, persistent AF, and spontaneous echo contrast in transesophageal echocardiography. Significant procedural parameters were electric cardioversion, PVI only, and ablation of complex atrial electrograms. Independent risk factors in multivariate analysis were age, spontaneous echo contrast and ablation of complex atrial electrograms.

Previous studies also confirmed that procedural ACT and performing cardioversion during the procedures were the risk predictors of post-AF ablation SCEs (26, 35). ACT levels of >300 s have been recommended to better reduce thromboembolic risk (36, 37). A 2.75-fold increase in the risk of subclinical cerebral embolism was related to periprocedural cardioversion (26). Pianelli et al. (38) suggested that delaying electrical cardioversion until after a 4-week anticoagulation period could reduce the risk of SCEs and is a viable and safer option in patients for which the ablation procedure does not result in acute termination of AF. However, the association between brain lesions and periprocedural cardioversion is not fully resolved as evident in other studies (20, 39, 40). In our study, ACT was maintained at >250 s and one-half of patients underwent cardioversion in the RMN group.

Although the percentage of patients requiring cardioversion was much higher in the MC group, our finding is that ACT and cardioversion are not major contributors to SCEs in this study.

Another important risk factor of SCEs is AF ablation technology. Different technologies can have varying effects on the incidence of SCEs. The incidence of SCEs after AF ablation may change according to the technology used (25). Herrera Siklódy et al. (9) compared the safety of different devices by screening for SCEs after AF ablation with either conventional irrigated radiofrequency, cryoballoon, or multielectrode phased radiofrequency pulmonary vein ablation catheter (PVAC). The incidence of SCEs in the irrigated radiofrequency group, cryoballoon group and PVAC group were 7.4, 4.3, and 37.5%, respectively. In another study, the incidence of SCEs after AF ablation differed depending on the technology used: PVAC increased the risk of SCEs 1.48 times compared to irrigated radiofrequency and cryoballoon ablation (25). It is currently clear that the prevalence of SCEs after AF ablation is related to ablation technologies (9, 22, 35, 41, 42), and therefore in this study, we investigated the incidence of SCEs in AF patients using RMN.

Analyzing the baseline clinical characteristics of the study population, we found that there were significant differences in age, CHA_2_DS_2_-VASc score, cardioversion, left atrial diameter, ejection fraction, NYHA heart function class and CVA/TIA history between two groups (Table 1), but when we compared the characteristics of patients with and without SCEs, we found that there were only statistical differences in CHA_2_DS_2_-VASc score, ejection fraction and CVA/TIA history between the two groups, though the range of ejection fraction in both groups was approximately 55–65%, which is normal, the clinical significance of this value is questionable (Table 2). To investigate the potential risk factors of SCEs, we first performed univariate logistic regression which indicated that ablation technology, CHA_2_DS_2_-VASc score, CVA/TIA history and low ejection fraction were significantly associated with SCEs. In a multivariate logistic regression analysis model, when ablation technology, CHA_2_DS_2_-VASc score, ejection fraction and CVA/TIA history were included as the controlling variables, we found that ablation technology (MC ablation) was the only independent risk factor of SCEs, with an odds ratio of 7.778.

### Mechanisms of Silent Cerebral Embolism

In 2003, SCE was first reported in patients with valvular aortic stenosis who underwent retrograde catheterization of the aortic valve and had 22% silent ischemic brain lesions (43). In 2006, SCE was first reported in patients undergoing AF ablation (7). In the years that have passed, the mechanisms of SCE associated with AF ablation are not yet fully understood (27, 44). The genesis of SCE appears to be multifactorial (24), and in recent years, several probable embolic sources have been considered such as: (1) gaseous emboli entrapped within the sheath during catheter insertion or extraction; (2) particulate emboli (so-called char) as a result of denaturation of tissue; (3) regional micro-thrombi formed at the ablation site; (4) micro-thrombi inside the sheath or other artificial instruments; and (5) gaseous emboli formed during heating of blood.

In 2011, Boersma (45) commented “no bubbles, no troubles” when reflecting on the mechanism of SCEs after AF ablation, suggesting that if no bubbles appeared during an AF ablation procedure, then there would be no SCEs (troubles). Nagy-Balón et al. (46) compared the occurrence of bubble formation seen on intracardiac echocardiography and the microembolic signals detected by transcranial Doppler on the use of different ablation technologies and found that most of these microemboli are gaseous in nature. Previous animal studies also have demonstrated that direct arterial injection of gaseous material similar to that generated by AF ablation can recreate SCEs seen in patients post-procedure (44). The second largest source of gaseous emboli in the animal model was found to be introduction of air into the left atrium via the sheaths during catheter insertion and removal (40). Air microemboli may be introduced into the blood stream through sheaths and catheters or may develop during ablation as a result of blood boiling during AF ablation (42).

Thrombus formation during and after AF ablation might result from platelet and coagulation system activation either directly at the catheter surface or at the site of endothelial application (42). The exact mechanisms of SCEs after AF ablation are unknown, as is the precise composition of the microemboli which generate microembolic signals. The possibilities include thrombus, coagulum/char, air, or steam (47). Risk factors associated with increased incidence of SCEs involve patient-specific, technology-associated and procedural determinants (25). It is currently clear that the prevalence of SCEs following AF ablation is related to ablation technology (9, 22, 38, 39).

### Possible Mechanisms of Decreased Incidence of SCE in AF Ablation Using RMN

The exact mechanisms of SCEs after AF ablation are still unknown, and we do not yet know the precise composition of the microembolic signals (47). However, it is commonly accepted that the increased thrombotic risk is due to intraprocedural introduction of gaseous microbubbles into the left atrium, thrombus formation within the lumens of long sheaths, char formation on the ablation catheter tip, and the potential for thrombus formation on the ablated atrial endocardium (48). Furthermore, the technology used for ablation does seem important as demonstrated by phased radiofrequency ablation more often resulting in observing abnormalities on cerebral MRI (25, 26), however, AF ablation using a novel gold tip resulted in SCEs in approximately only one out of 10 patients (49). The mechanisms of decreased incidence of SCEs in AF ablation using RMN may be as follows:

#### AF Ablation Using RMN Reduces Gaseous Microbubbles

The study of Nagy-Baló indicated that 80% of the microemboli are gaseous, suggesting that gaseous microbubbles may be more prevalent during AF ablation procedures (47). More aggressive anticoagulation reduced but did not eliminate the occurrence of microembolic signals, supporting the hypothesis that thrombus only accounts for a minority of the particulate embolic burden (46). Air embolisms are the main mechanism of SCEs, which has also been confirmed after second-generation cryoballoon ablation procedures (50). Air embolisms may be related to the introduction of ablation devices (51, 52). Multiple intraprocedural exchanges of catheters have a major impact on the risk for SCEs. When exchanging catheters in transseptal sheaths, gaseous emboli can be entrapped within the sheath during catheter insertion or removal.

Regarding AF ablation using RMN, when an AF ablation procedure is performed, only one Swartz sheath is inserted into the right femoral vein, and fixed on a computer-controlled catheter advancing system (QuikCAS, Niobe Stereotaxis, St. Louis, MO, USA). There is no relative movement between the sheath and the femoral vein, and no exchange of catheter during the entire AF ablation procedure. However, a double transseptal puncture is needed and two Swartz sheaths are placed in the left atrium when MC ablation is performed. Multiple catheter exchanges to facilitate the catheter ablation of pulmonary vein isolation during a MC ablation procedure may introduce gaseous microbubbles to the left atrium, which could lead to greater SCE formation.

#### Single Sheath in Left Atrium Reduces Clot Formation

In addition to air/gas embolism, clot formation is one of the main causes of SCE origin during AF ablation (53). Many studies have confirmed that the more sheaths or other artificial instruments in the left atrium, the more micro-thrombi will appear, leading to more SCE formation. When AF ablation using RMN is performed, only one Swartz sheath and one ablation catheter enter the left atrium (14). However, when MC ablation is performed, a double transseptal puncture is utilized, and two Swartz sheaths and other mapping catheters like a Lasso or Pentaray catheter (Biosense Webster, Inc., Diamond Bar, CA) are placed in the left atrium. Micro-thrombi may be formed inside the sheaths or other artificial instruments.

#### Uniquely Flexible RMN Catheter Reduces Char Formation and Complications

Microparticles, so-called char resulting from tissue denaturation, are also one of the main causes of SCE origin during AF ablation (27, 40, 53). Char formation may be induced by the energy source and / or catheter tip pressure on the tissue (53). Techniques and technologies to reduce excess char embolism have helped to reduce SCE prevalence, suggesting that char formation is a common perpetrator (47).

The RMN catheter's uniquely flexible shaft provides increased safety as well as equivalent or superior efficacy (54, 55). These results are achieved due to the catheter's atraumatic design, superior reach, and tip stability on the myocardium.

Bhaskaran et al. (55) performed an experimental study to compare lesion dimensions and evaluate the effect of heart wall motion in a myocardial phantom using RMN vs. MC. They found that similar lesion dimensions were observed in the stationary model; however, the lesion dimensions were more focal and deeper with RMN compared to MC in the presence of simulated wall motion, demonstrating greater catheter stability in the RMN group. During motion, the manual catheter slid across the surface 5.5 mm while the magnetic catheter maintained stable focal contact. A sliding catheter requires more total energy delivery to achieve an effective lesion at the target site. Therefore, the RMN catheter's stability may be a factor in reducing tip char formation during AF ablation procedure.

### Study Limitations

There are at least three limitations in this study. First, this study is a prospective but non-randomized trial. However, between the two groups there were few significant differences in either the baseline characteristics or the characteristics of patients with SCEs and without SCEs. Second, this novel research was designed to be a pilot study analyzing the incidence of SCEs using RMN and thus contributing important information regarding the effect of ablation technologies and techniques on SCEs. The incidence of SCEs in the RMN group is significantly lower than in the MC group, however, the results must be interpreted acknowledging that the sample size was smaller in the MC group, and therefore a larger, multi-center, prospective study may be warranted. Finally, the exact mechanisms of decreased SCEs using RMN ablation remain elusive. The three main mechanisms we provided in the discussion are the probable mechanisms, and the use of intracardiac ultrasound in further studies may help us to delineate more exact mechanisms.

## Conclusions

AF ablation carries a low risk of symptomatic cerebral ischemia but is associated with a substantial risk of SCEs. Ablation technology, CHA_2_DS_2_-VASc score, CVA/TIA history, and low ejection fraction are associated with SCEs after AF ablation procedure. However, ablation technology is the only independent risk factor of SCEs and RMN can significantly reduce the incidence of SCEs resulting from AF ablation.

## Data Availability Statement

The original contributions presented in the study are included in the article/supplementary material, further inquiries can be directed to the corresponding author/s.

## Ethics Statement

The studies involving human participants were reviewed and approved by 1. Wuxi People's Hospital Affiliated to Nanjing Medical University; 2. Xuzhou Central Hospital; 3. First Hospital Affiliated to Soochow University. The patients/participants provided their written informed consent to participate in this study.

## Author Contributions

RXW, YY, BH, and TBJ contributed to the design of study, analysis, and interpretation of data for the publication, writing of the manuscript, and the final approval of the manuscript. KLL, XYL, and XXZ contributed to performing the procedures of atrial fibrillation. QFT, CYZ, and ZMY performed the analysis of MRI images. MW and SPD contributed to the acquisition and statistical analysis of data for the study. All authors contributed to the manuscript revision and reading and approved the submitted version.

## Funding

This study was supported in part by grants from the National Natural Science Foundation of China (81770331), Natural Science Foundation of Jiangsu Province (BK20151110), and Chinese Cardiovascular Association V.G foundation (grant number: 2017-CCA-VG-040).

## Conflict of Interest

The authors declare that the research was conducted in the absence of any commercial or financial relationships that could be construed as a potential conflict of interest.

## Publisher's Note

All claims expressed in this article are solely those of the authors and do not necessarily represent those of their affiliated organizations, or those of the publisher, the editors and the reviewers. Any product that may be evaluated in this article, or claim that may be made by its manufacturer, is not guaranteed or endorsed by the publisher.
