# Supplementary material for: Atrial Fibrillation Ablation Using Robotic Magnetic Navigation Reduces the Incidence of Silent Cerebral Embolism
Source: Front Cardiovasc Med. 2021 Dec 1;8:777355. doi: 10.3389/fcvm.2021.777355 (PMC8671737; doi:10.3389/fcvm.2021.777355)
Supplement: Supplementary file 1 [file Data_Sheet_1.PDF]

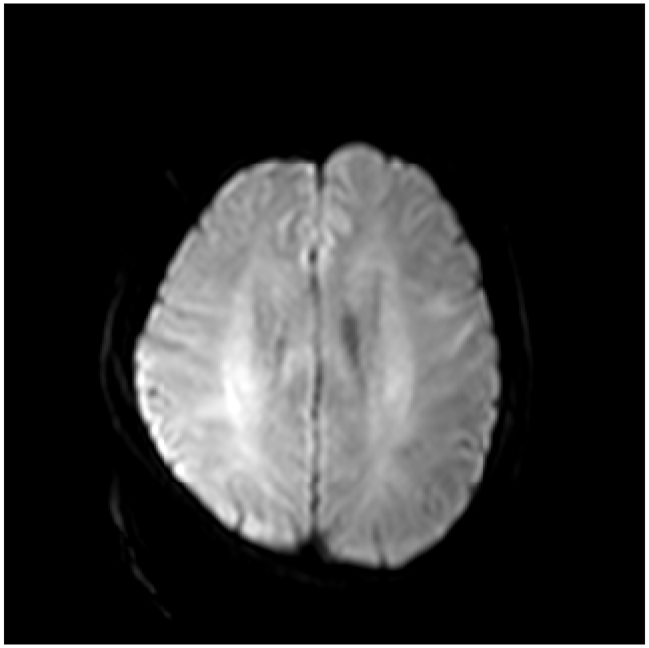

Pre-procedure

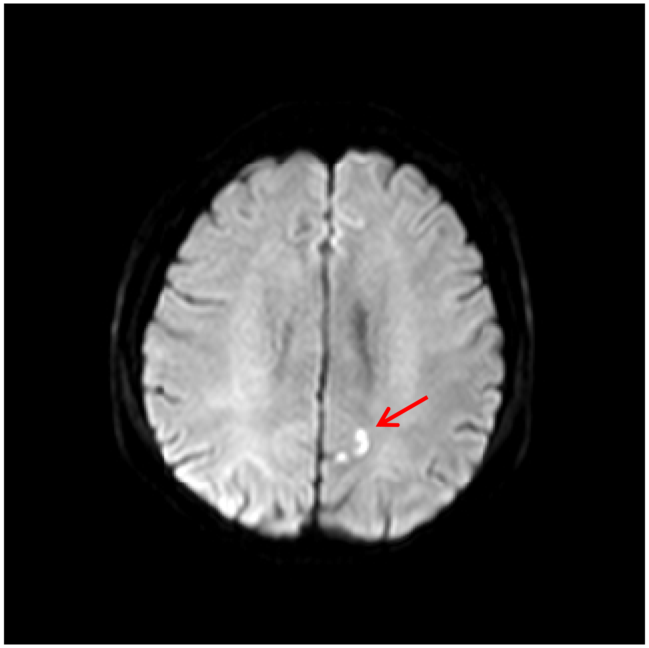

Post-procedure
(13mm)

Case 1

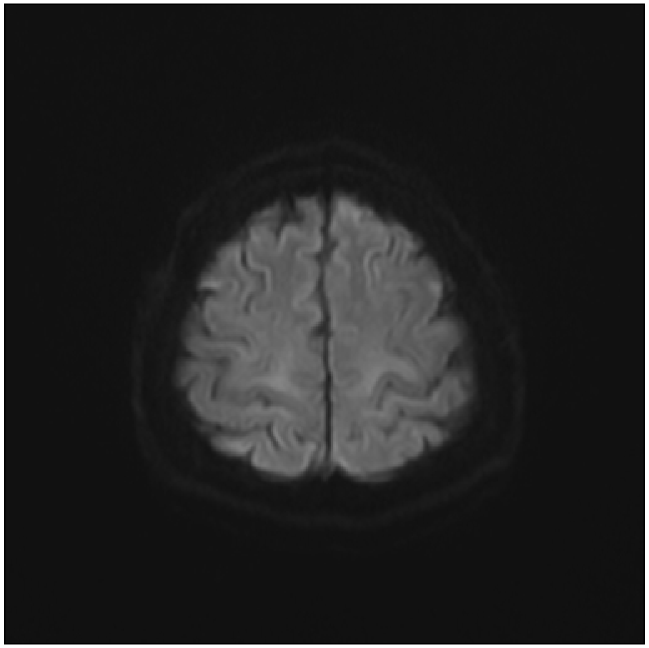

Pre-procedure

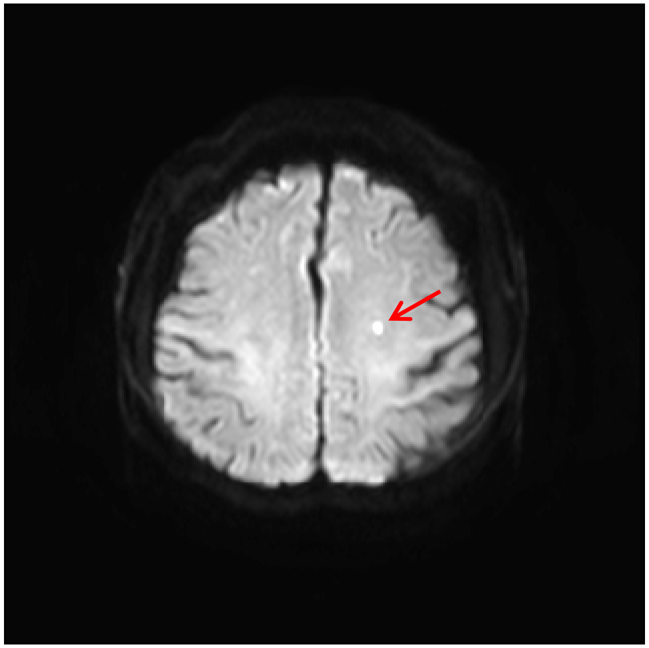

Post-procedure
(3.5mm)

Case 2

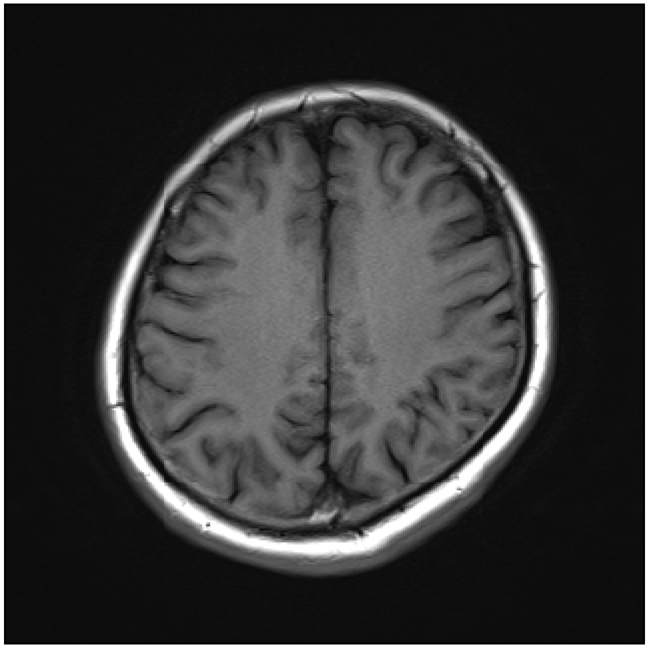

Pre-procedure

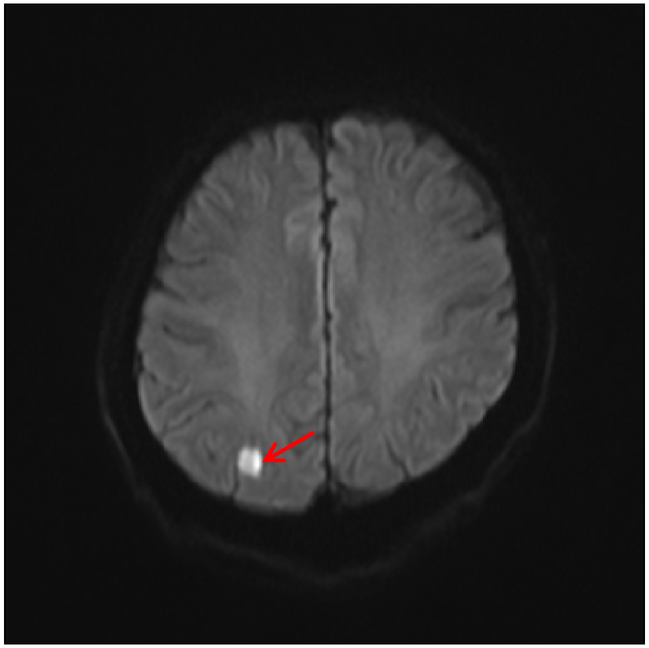

Post-procedure
(8.6mm)

Case 3

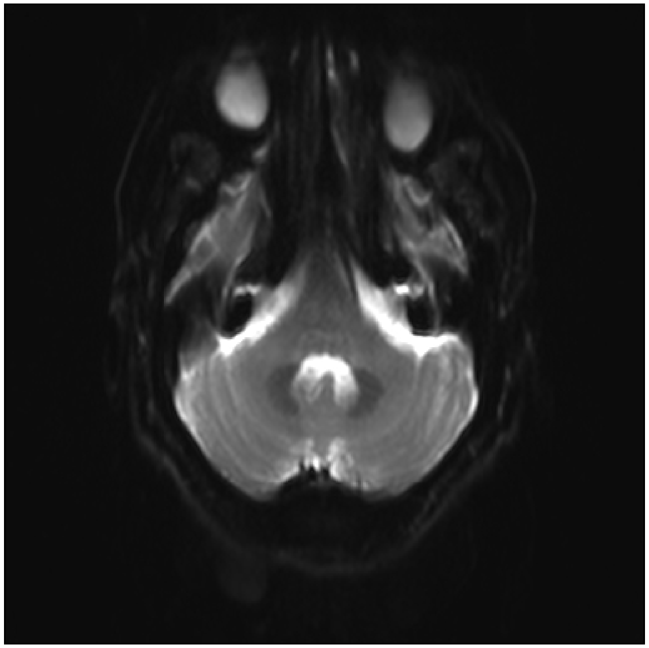

Pre-procedure

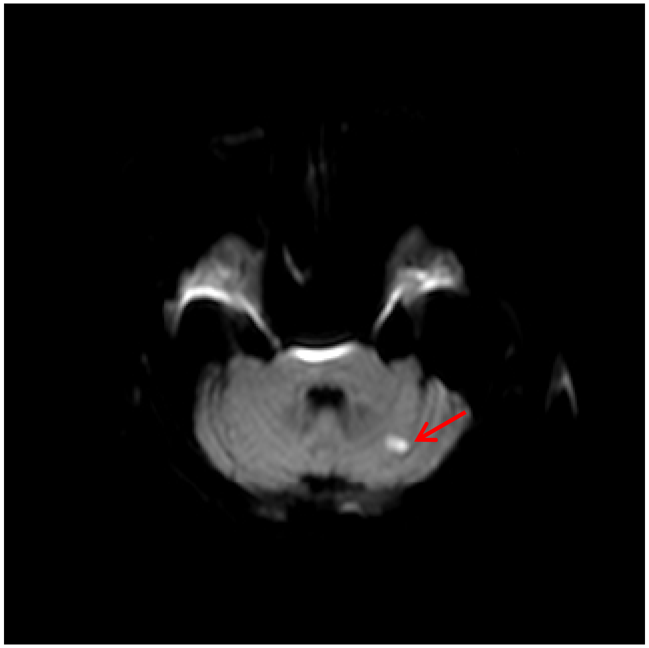

Post-procedure
(7.3mm)

Case 4

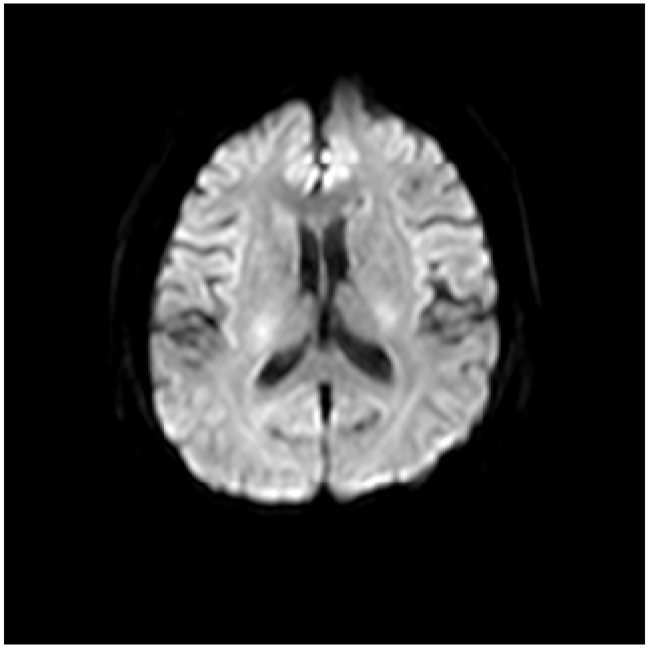

Pre-procedure

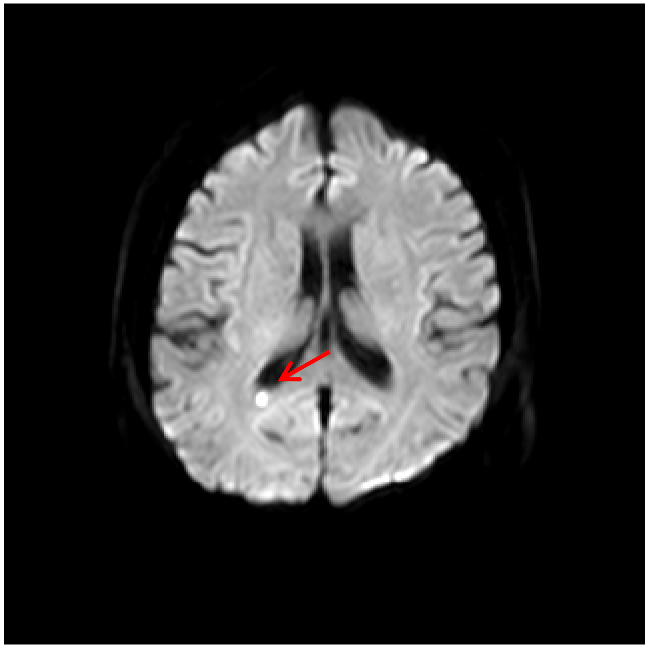

Post-procedure
(3.6mm)

Case 5

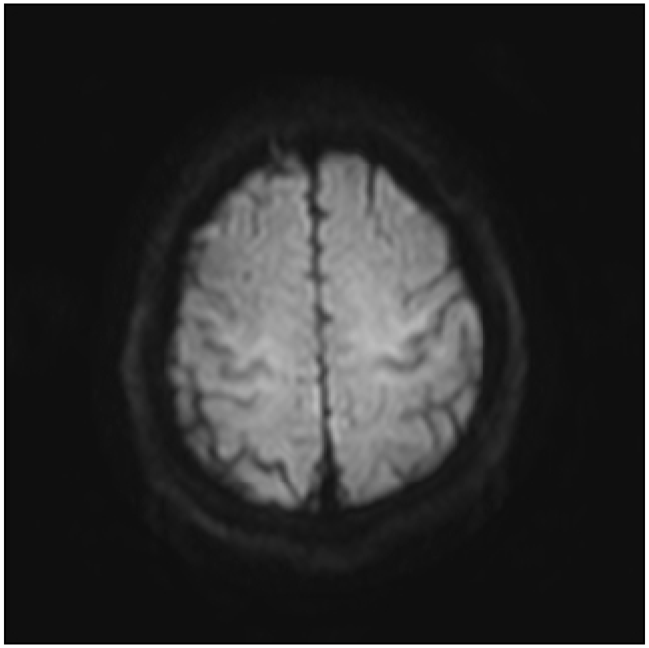

Pre-procedure

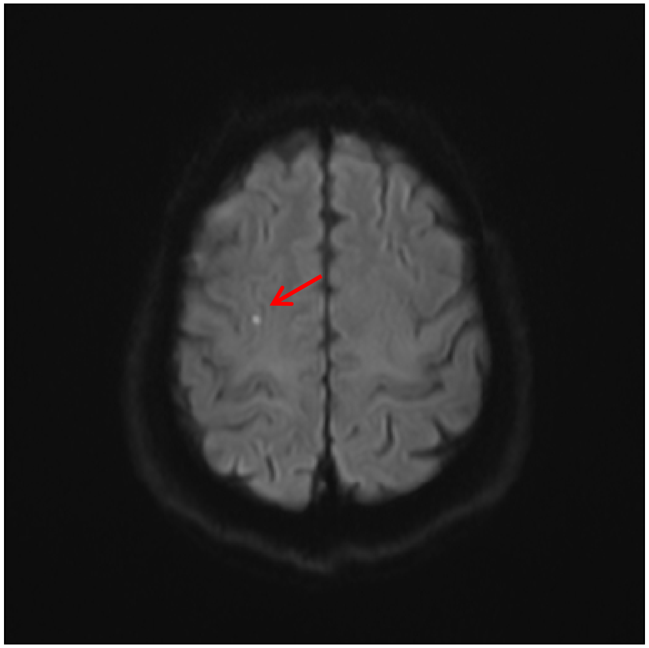

Post-procedure
(1.9mm)

Case 6

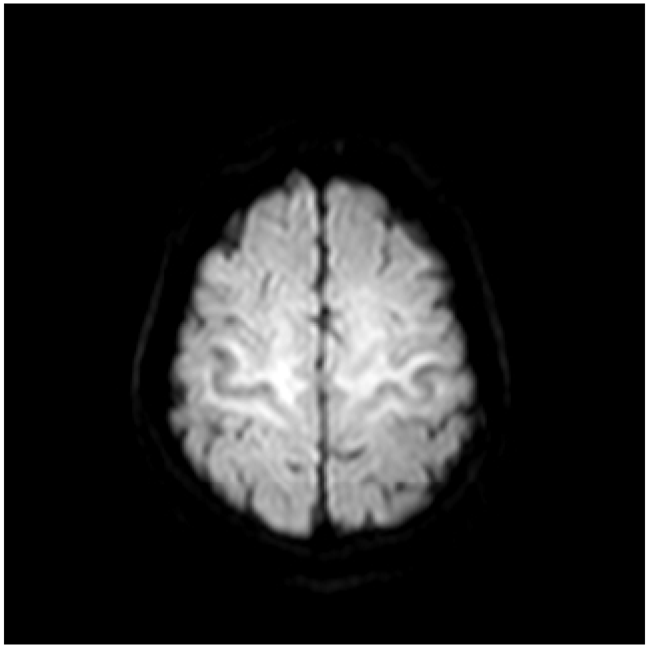

Pre-procedure

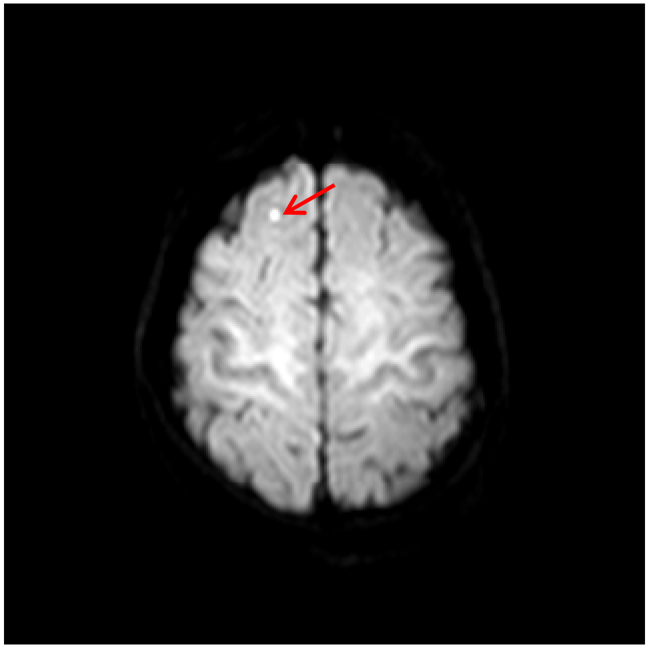

Post-procedure
(3.6mm)

Case 7

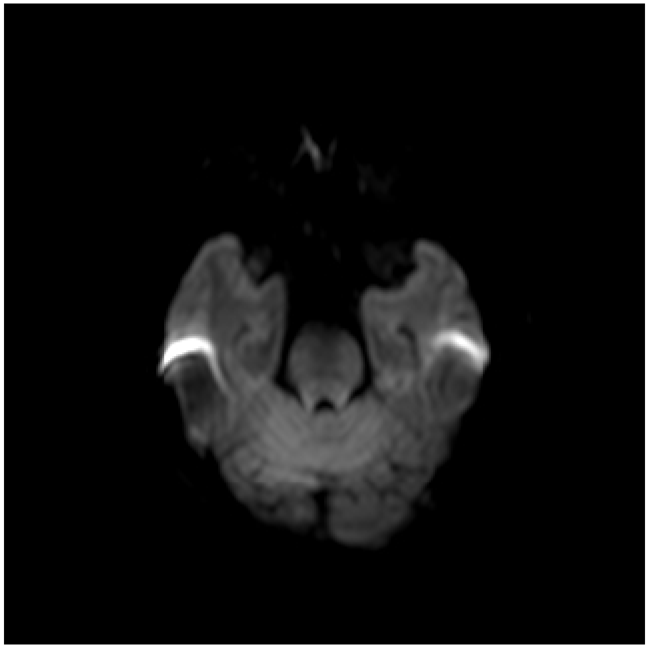

Pre-procedure

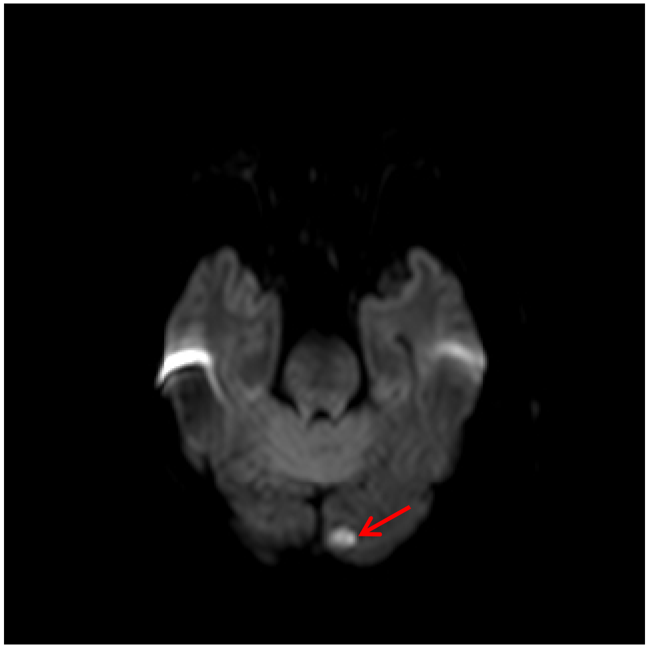

Post-procedure
(9.3mm)

Case 8

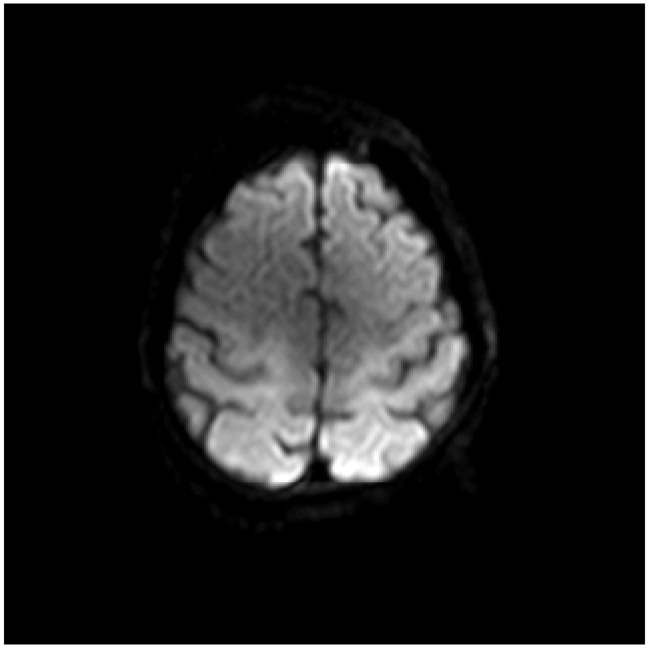

Pre-procedure

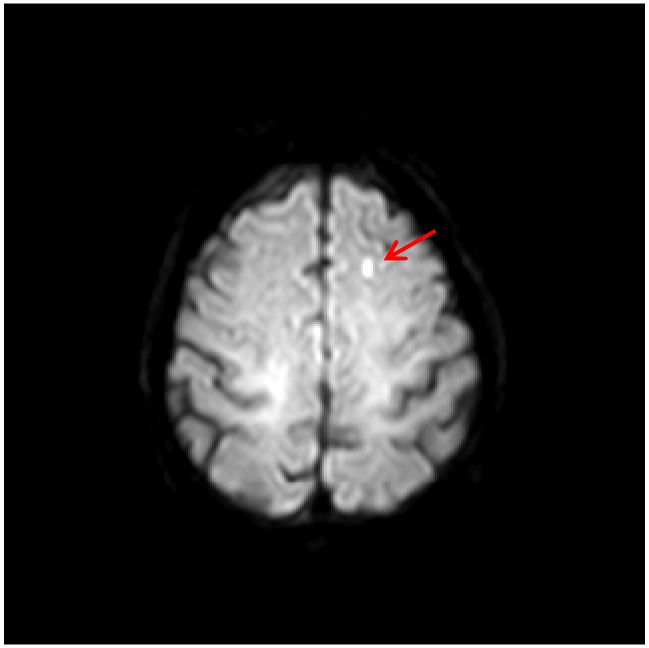

Post-procedure
(6.5mm)

Case 9

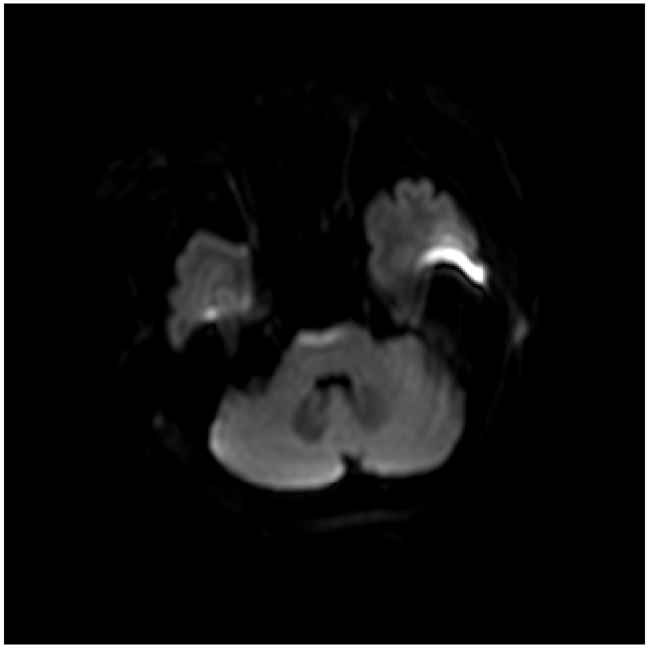

Pre-procedure

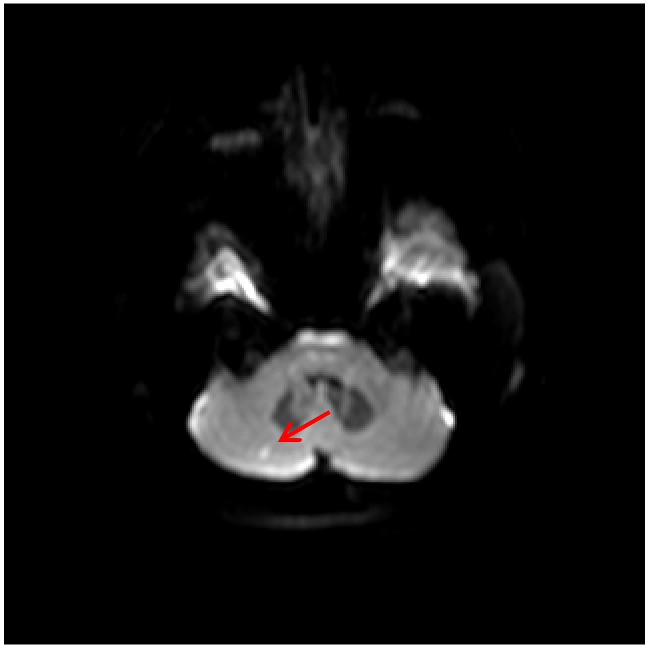

Post-procedure
(4.2mm)

Case 10

Pre-procedure

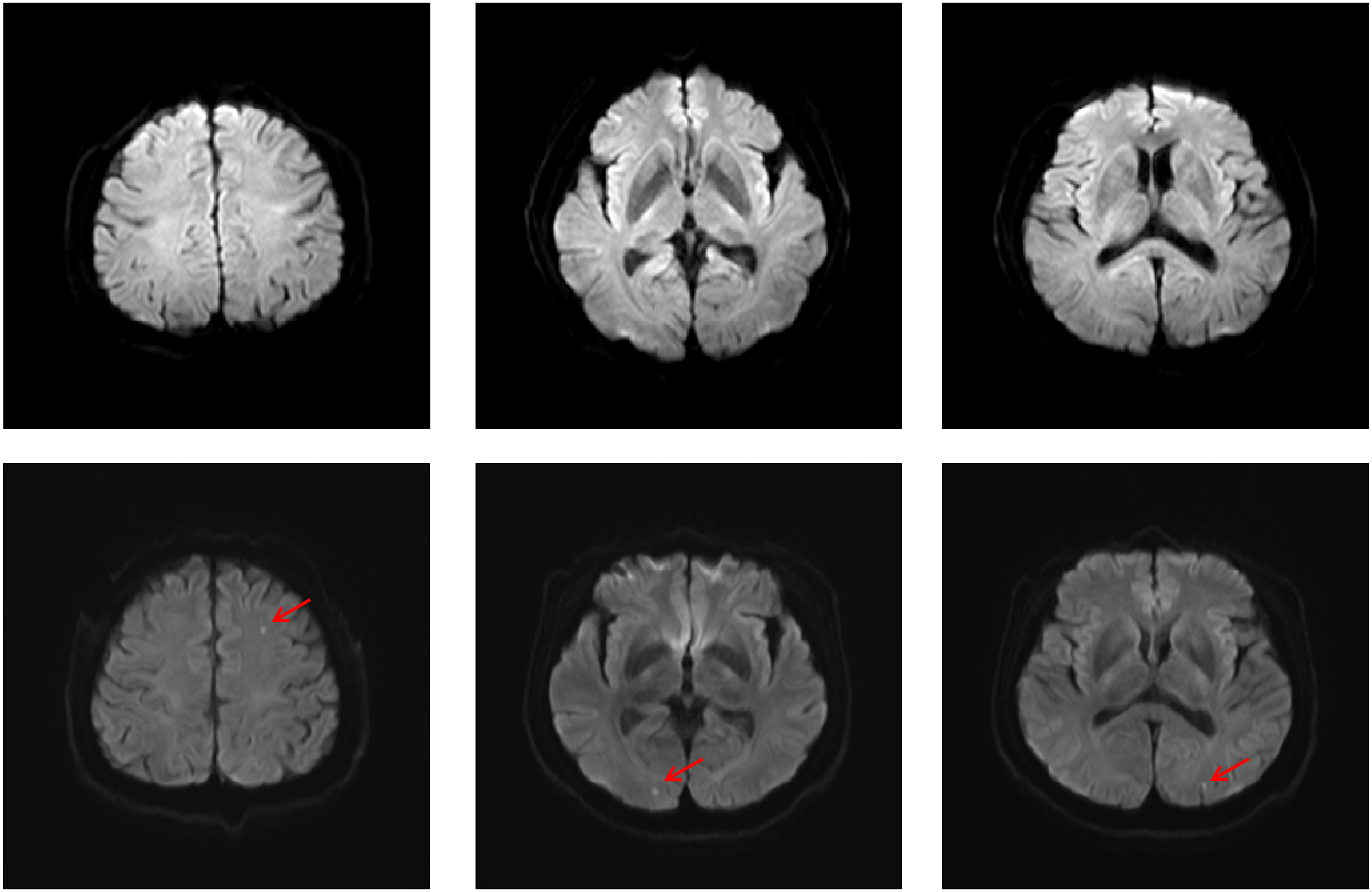

Post-procedure (2.9mm)

Post-procedure (2.9 mm)

Post-procedure (2.6mm)

Case 11

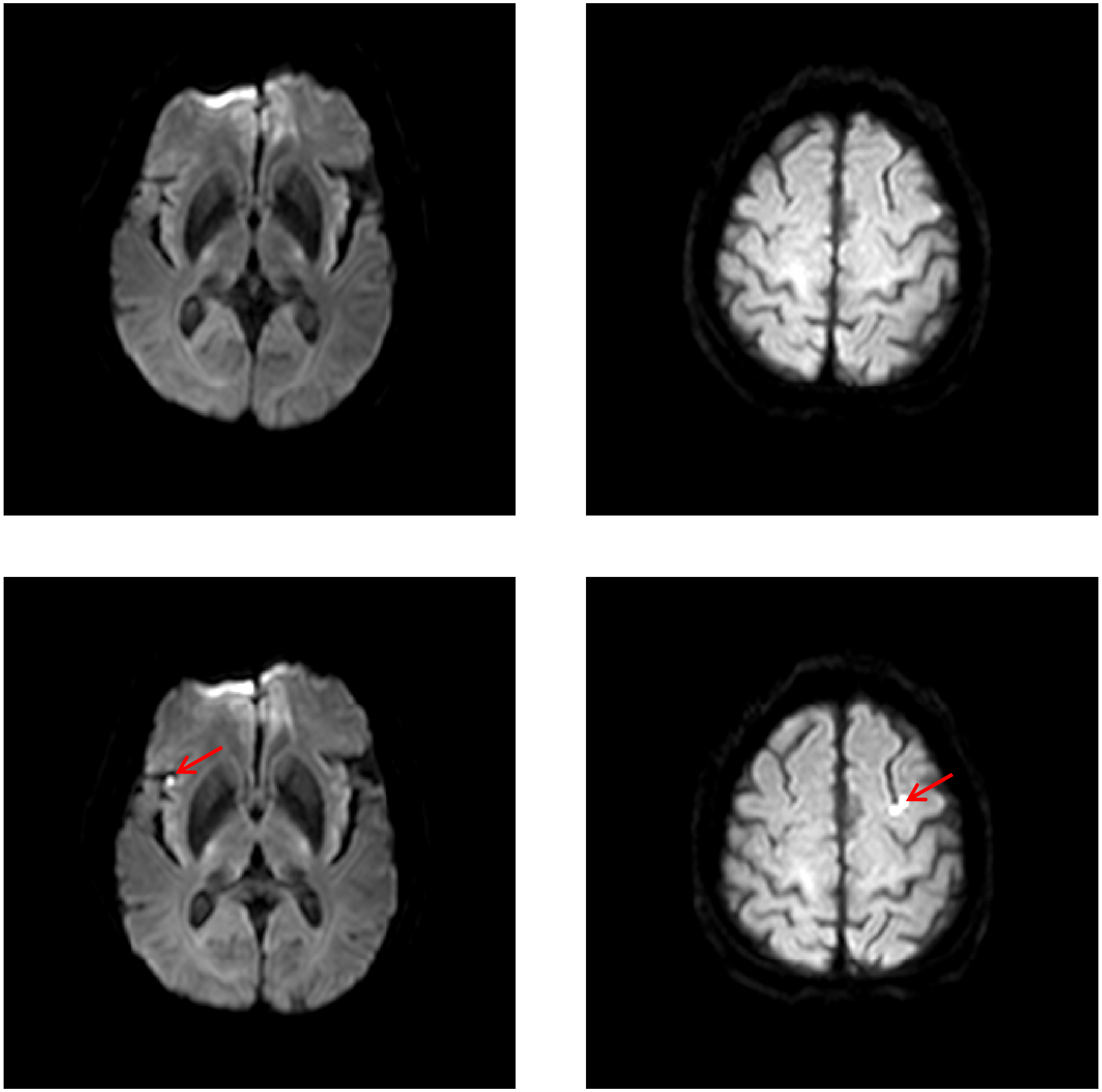

Pre-procedure

Post-procedure

2.8mm

9.6mm

Case 12

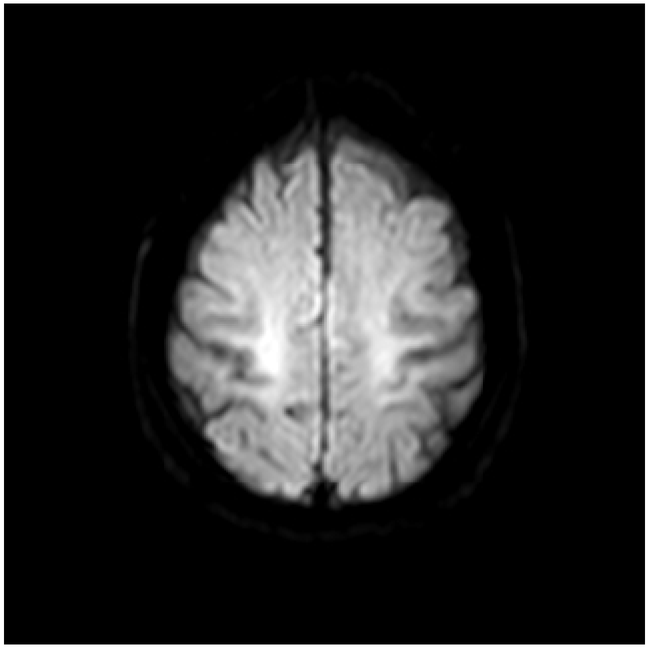

Pre-procedure

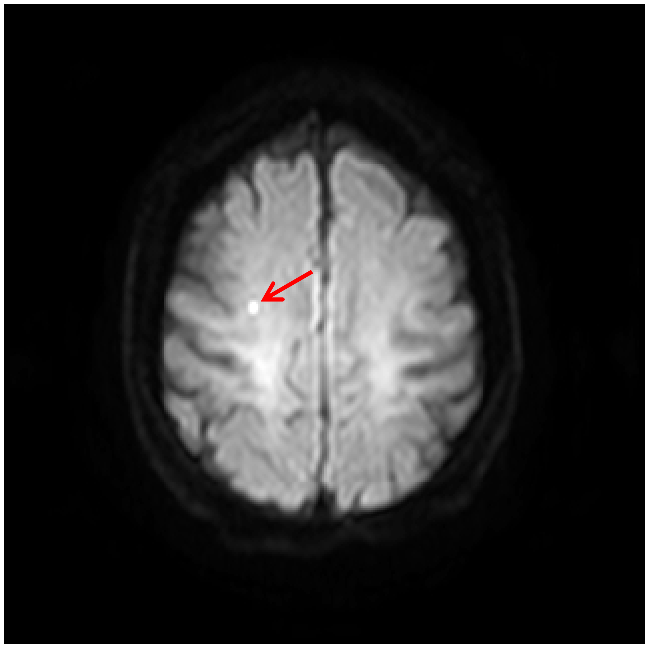

Post-procedure
(4.5mm)

Case 13

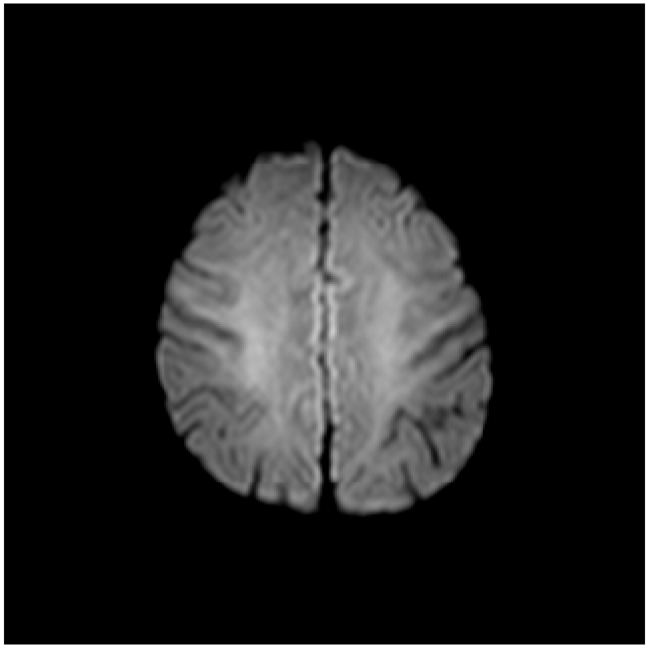

Pre-procedure

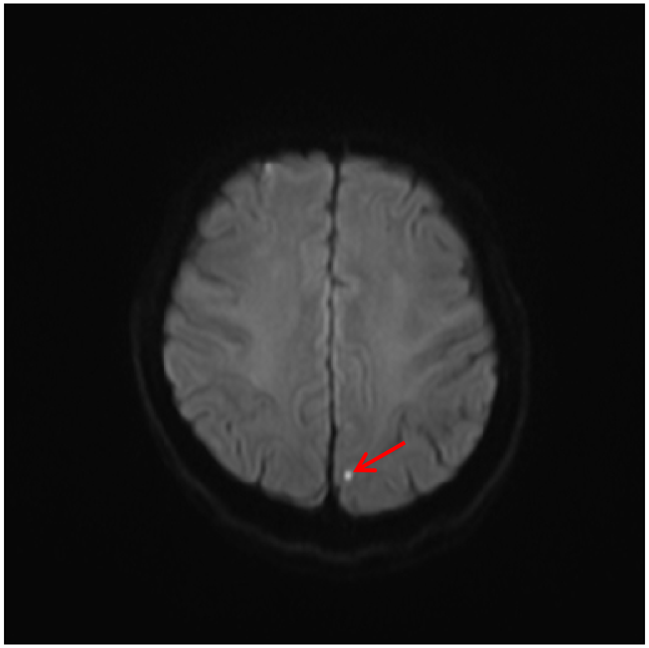

Post-procedure
(3mm)

Case 14

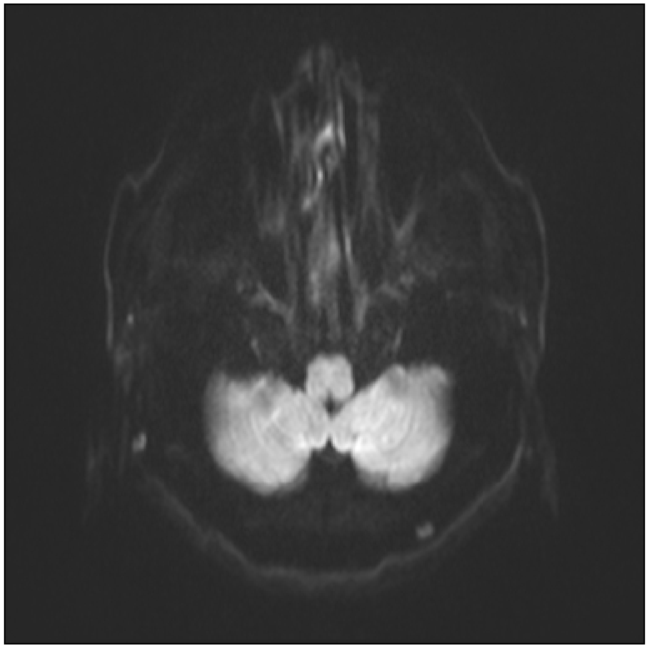

Pre-procedure

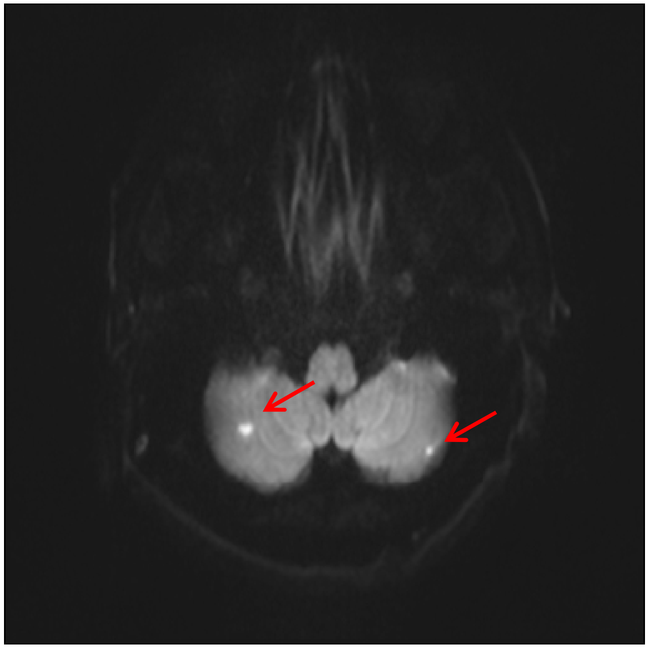

Post-procedure
(4.2mm；2.4mm)

Case 15

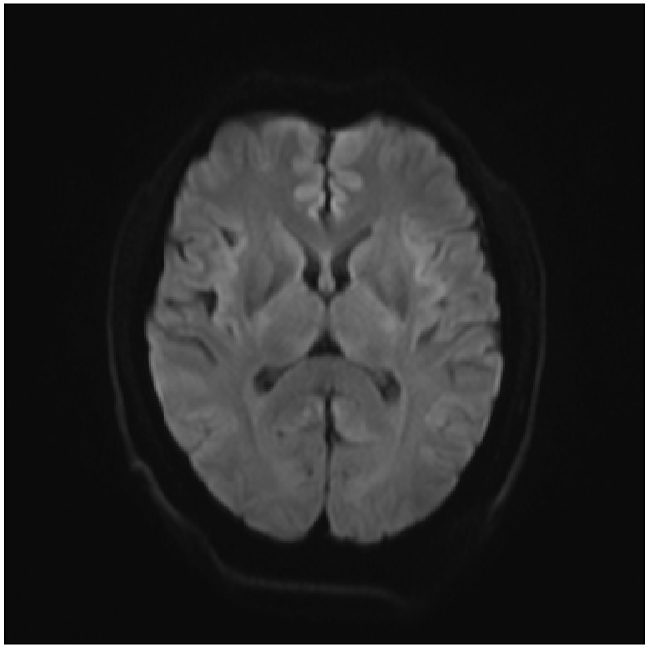

Pre-procedure

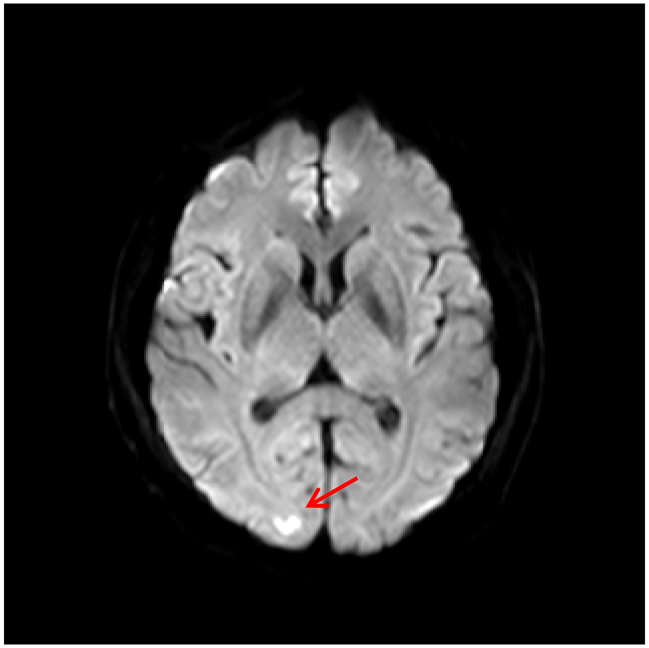

Post-procedure
(9.3mm)

Case 16

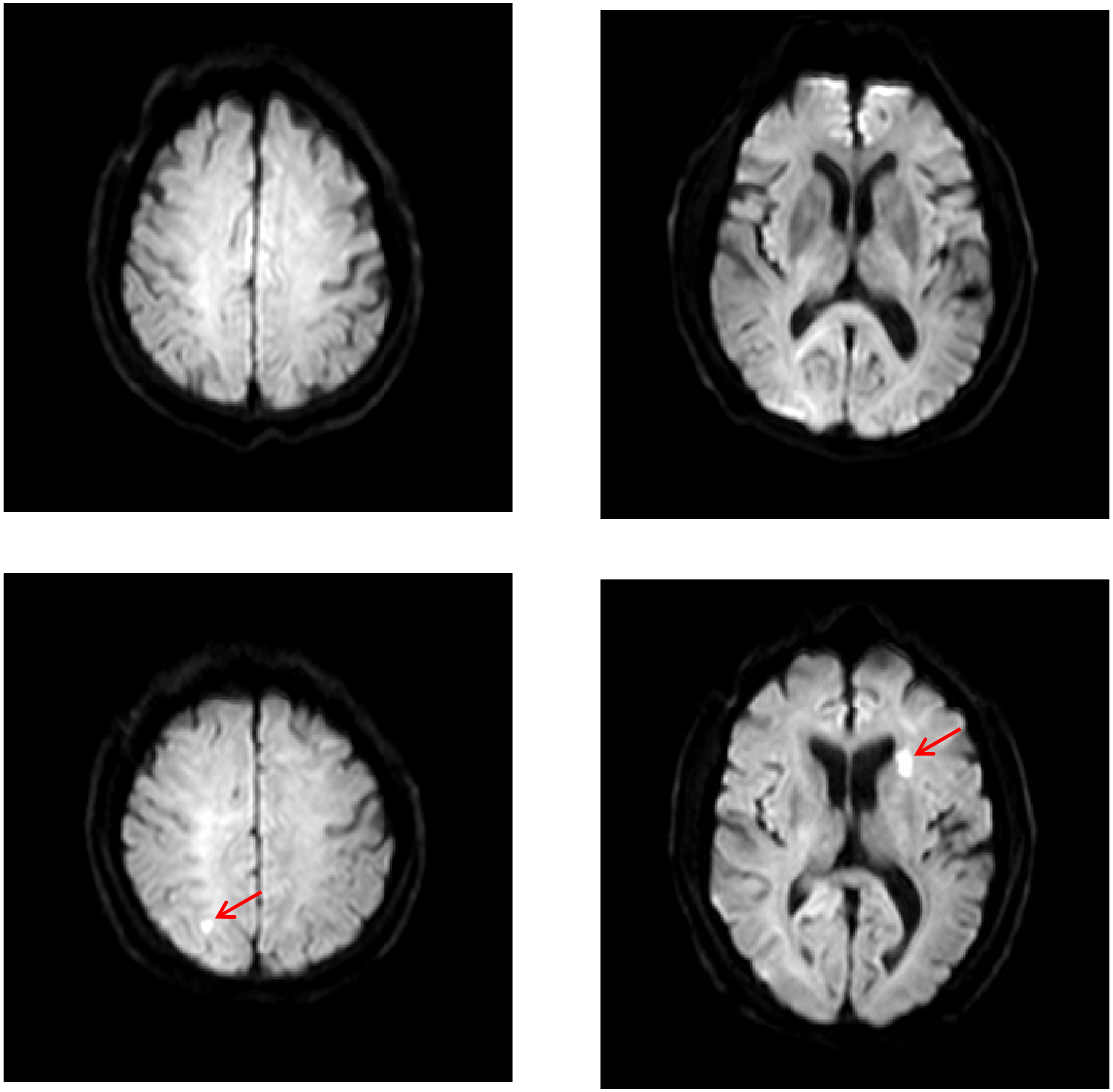

Pre-procedure

Post-procedure
(5.2mm)

Post-procedure
(10.8mm)

Case 17

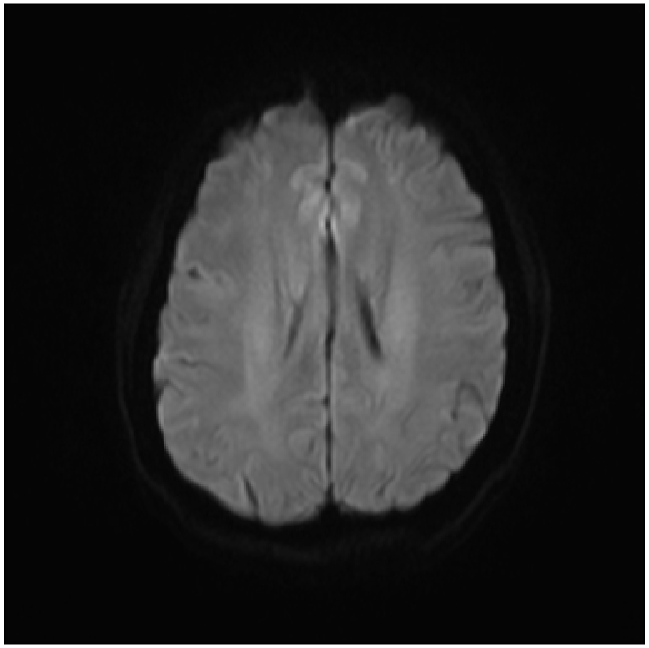

Pre-procedure

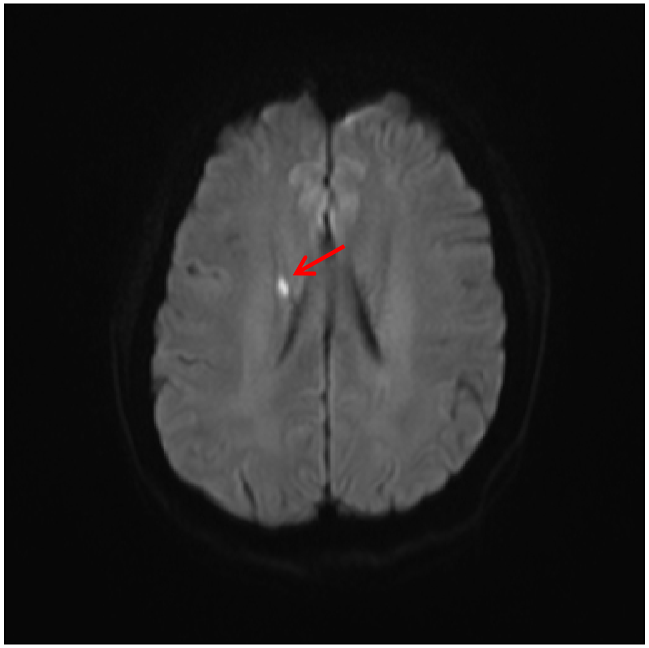

Post-procedure
(5mm)

Case 18

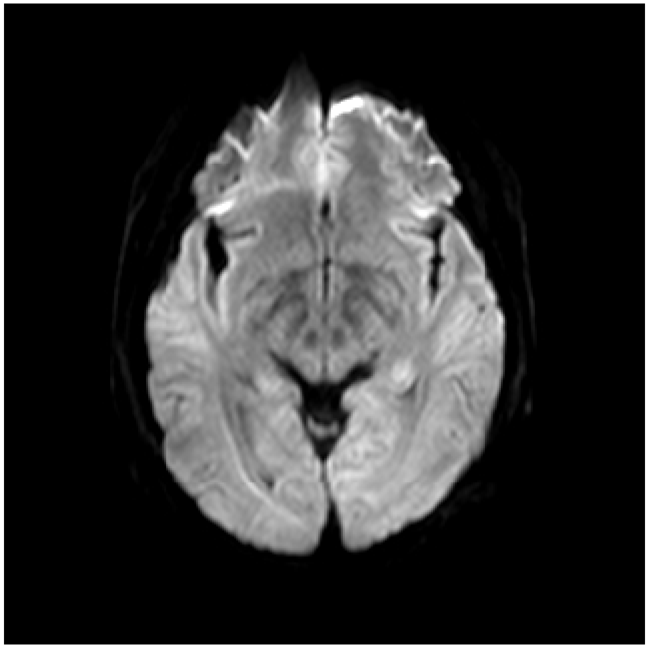

Pre-procedure

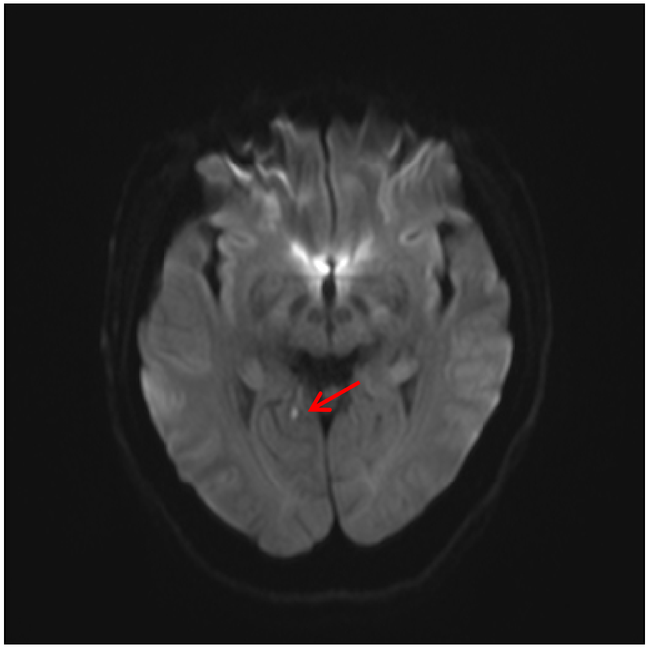

Post-procedure
(3mm)

Case 19

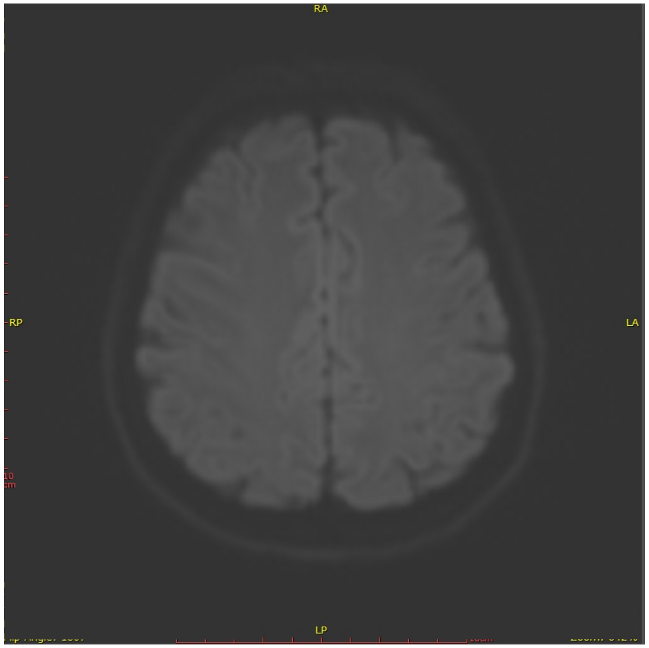

Pre-procedure

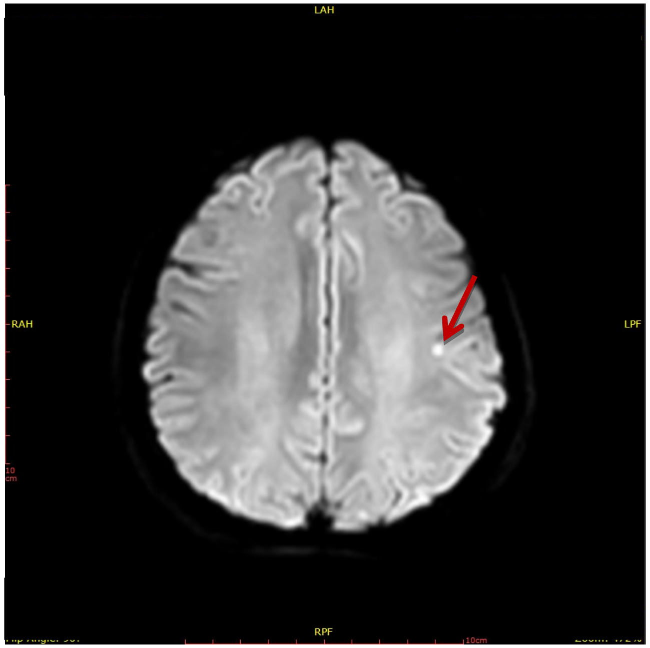

Post-procedure
（4mm）

Case 20
